# Oculopharyngeal muscular dystrophy misdiagnosed as myasthenia gravis: Case report and review of literature

**Published:** 2017-04-04

**Authors:** Omid Aryani, Mohammadreza Akbari, Masoud Aghsaei-Fard, Arash Mirmohammad-Sadeghi, Samira Yadegari

**Affiliations:** 1 Department of Medical Genetics, Special Medical Center, Tehran, Iran; 2 Department of Neuro-Ophthalmology, Eye and Strabismus Research Center, Farabi Eye Hospital, Tehran University of Medical Sciences, Tehran, Iran

**Keywords:** Ptosis, Oculopharyngeal Muscular Dystrophy, Myasthenia Gravis, Diagnosis

Oculopharyngeal muscular dystrophy (OPMD) is an adult-onset disease with eyelid ptosis, progressive dysphagia, and proximal limb weakness. Despite recent advances in understanding of its molecular basis, it seems that OPMD remains under diagnosed or delayed in diagnosis.^1^ This could be due to slow progression of this disease, low prevalence of 1:100000^2^ or low attention of neurologists that may diagnose and treat the disease as other neuromuscular disorders. Herein, we present a case of genetically approved OPMD in which the patient underwent unnecessary therapeutic intervention due to misdiagnosis of myasthenia gravis (MG) and then in regard to this case, explain some clinical clues to make the appropriate diagnosis.

A 55-years-old man known case of MG from eight years ago was referred to us for plasmapheresis due to progressive difficulty in swallowing. He had history of ptosis for the last eight years with no complain of diplopia. His dysphagia had been begun since three years ago. Thymectomy was performed for him three years ago via thoracotomy with normal thymus pathology. He denied any positive family history of similar disease. His medications contained mestinon, azathioprine 150 mg and prednisolone 15 mg/day for the last 6 years. His neurologic exam revealed bilateral moderate to severe ptosis, bifacial paralyses, and ophthalmoplegia on both eyes. Neck flexion and extension were 4/5. Motor strength had decreased in proximal of lower limbs (4/5). Previous laboratory evaluations consisted of a negative serum acetylcholine receptor antibody (< 0.1 nmol/l) and low frequency repetitive nerve stimulation (RNS) with 10% decrement on trapezius muscle. Creatine phosphokinase (CPK) was normal. Electrophysiological studies were repeated that showed chronic myopathic process and 3-Hz RNS revealed no reproducible decremental response. Regarding the patient's clinical history and disease course, we discouraged him for plasmapheresis and set an appointment where his sister and parents were present. We observed bilateral ptosis and bulbar speech in his 45-year-old sister and their mother. Molecular genetic analysis was performed. The poly-(A) binding protein nuclear-1 (PABPN1) DNA fragment flanking the (GCG)n(GCA)n repeat was amplified and a mutated allele (GCG)6(GCA)(GCG)4(GCA)3GCG was identified as compared to (GCG)6(GCA)3GCG in healthy subjects of PABPN1 gene.

OPMD is a degenerative, predominantly autosomal dominant disorder. The disease is characterized by a mutation in PABPN1 gene, resulting in a short GCG expansion in the polyalanine tract of PABPN1 protein.^1^ Its clinical features may have many similarities with MG. Both of them characterize with ptosis, extraocular muscle weakness, dysphagia, and limb weakness. The distribution of the affected muscles in OPMD largely overlaps with the muscles involved in MG and muscle biopsy may be non-specific.^3^ Moreover, fluctuating course in OPMD simulating MG is also reported.^3^ We described a genetically confirmed case of OPMD who was managed as a seronegative MG for ten years. He had been underwent a major thoracic surgery (thymectomy) and received immunosuppressive agents for long years. Regarding these unnecessary interventions, several points should be considered in clinical practice when dealing with the patients complaining of ptosis, ophthalmoparesis, and dysphagia. First, attention to the patient's clinical history is the main key of correct diagnosis. Fatigability and diurnal fluctuations is hall mark of MG versus insidious and slow progression in OPMD. Second, positive family history of patients with OPMD may be overlooked due to slowly progressive nature of the disease as in our patient. In a study of 14 patients with OPMD only six patients could report a family history of ptosis.^1^ Thus, visiting patient's family members or their photos could be helpful. 

The next important point is the limitations of laboratory studies and possible technical errors. Autoantibodies against the postsynaptic nicotinic acetylcholine receptor can be detected in the serum of 50% of patients with ocular MG. The low-frequency RNS test is of great value in the diagnosis of MG, but its sensitivity is low in ocular MG. In a study of patients with ptosis or ophthalmoparesis due to MG, RNS had sensitivity of 61%, specificity of 83%, positive predictive value of 79%, and negative predictive value of 68%.^4^ Furthermore, the diagnostic yield of RNS in different muscles could be varied. Therefore, the results of these tests, especially in borderline circumstances should be interpreted with caution. The course of our patient suggests that when a suspected patient of MG is unresponsive to different therapeutic interventions, reevaluation of patient and considering other neuromuscular differential diagnosis are logical before considering patient as a refractory case. In addition, the possibility of addition of a secondary disorder should also be considered as occurrence of OPMD in a seropositive patient with MG was reported.^5^

In conclusion, OPMD may be a great mimicker of MG, but often neglected. Awareness would improve diagnosis and preclude unnecessary and sometimes harmful therapeutic interventions.

## Notes:

**How to cite this article:** Aryani O, Akbari M, Aghsaei-Fard M, Mirmohammad-Sadeghi A, Yadegari S. Oculopharyngeal muscular dystrophy misdiagnosed as myasthenia gravis: Case report and review of literature. Iran J Neurol 2017; 16(2): 98-9.
